# The prevalence of the metabolic syndrome in a farm worker community in the Boland district, South Africa

**DOI:** 10.1186/s12889-016-3973-1

**Published:** 2017-01-11

**Authors:** Maritza J. Kruger, Theo A. Nell

**Affiliations:** Department of Physiological Sciences, Stellenbosch University, Mike De Vries Building, Merriman Avenue, Stellenbosch, 7600 South Africa

**Keywords:** Metabolic syndrome, Prevalence, Anthropometry, Obesity

## Abstract

**Background:**

In South Africa, not much is known about MetS in farm working communities. This study aimed to describe the prevalence of the MetS in a farm working population from the Boland winelands district of the Western Cape, South Africa.

**Methods:**

A cross-sectional study was followed among farm workers (aged 20–60 years) from surrounding wine estates. The questionnaires used described socio-demographic status, ethnic background, alcohol consumption, smoking, exercise and daily medication. Anthropometric assessments were performed and blood pressure measurements taken prior to blood sampling for serum insulin, glucose and fasting lipogram profiles.

**Results:**

The prevalence of the MetS was higher in women (46.3 vs 29.3%). Both men and women in the MetS group had a significantly higher waist circumferences (WC; *p* < 0.001 for both), whilst higher glucose levels were only significantly higher in the women (*p* < 0.001). Correlations showed significant differences between body mass index (BMI), WC and waist to hip ratio (W:H) and the different MetS risk factors.

**Conclusions:**

The female population in this study showed higher prevalence rates for the individual risk factors and the MetS overall. There is an urgent need to develop culturally sensitive health promotion programs addressing risk factors for metabolic syndrome among farm workers.

## Background

The metabolic syndrome (MetS) is a cluster of risk factors that are mostly associated with people living with obesity and diabetes [1], with the prevalence of the MetS varying between different populations. Davila et al. [2] established that the prevalence of the MetS is not only increased in shift workers, but also among farm workers in the United States. One specific study from the African continent also highlighted urbanisation as a crucial factor in the prevalence of the MetS, especially in Sub-Saharan Africa [3]. South African data (CRIBSA) suggested a high prevalence among urban black populations [4]. This is mostly attributed to the nutrition transition [5] that contributes towards changes in dietary behaviour, social and psychological shifts. Although a limited number of studies are available regarding the prevalence of the MetS, not much is documented in the South African context. A further complication is the use of different MetS definitions. Here, it ranges from insulin resistance being reported to waist circumference (WC) not being reported. Motala et al. [6] reported an overall high prevalence of the MetS in rural women (30.2%) in South Africa. A study in an urban area in Cape Town revealed the prevalence of the MetS to be 60.6% (IDF definition) in 2012, and more associated with the coloured female population [7]. Age-adjusted prevalence of central obesity was also higher in women from rural (farming areas) *vs* urban areas [8]. There is also currently limited to no South African literature available on the prevalence of the MetS among farm workers. The aim of this study thus was to describe the prevalence of the MetS, as well as to highlight possible associations between selected parameters, in a farm worker population in the Boland district of the Western Cape, South Africa [9].

## Methods

### Study population

A cross-sectional study was followed among workers from the surrounding wine estates in the Stellenbosch Municipal area, Western Cape Province of South Africa. Meetings were held on each of the respective farms prior to study commencement at which time the study was thoroughly explained. Thereafter data was collected from volunteering participants of both genders that met the inclusion criteria from either Villiera-, Neethlinghshof wine estates, and Solms-Delta wine estate in the Franschoek Municipal area. Participants that were successfully recruited had to be from the Stellenbosch and surrounding area and also between 20 and 60 years old. Pregnant and/or lactating women were excluded at the time of data collection. Individuals that were unable to provide written consent were also excluded from the study.

### Data collection and questionnaires

All volunteers were informed of all testing procedures and what is expected from them. Information regarding socio-demographic status was obtained by means of a specifically designed questionnaire. The demographic questionnaire included questions regarding the number of people residing in the house, the type of house, home language and literacy, nearest clinic, and average monthly or weekly income. Specific questions regarding age, gender, ethnic background, alcohol consumption, smoking, exercise and daily medication were also included.

Conventional anthropometry was performed in duplicate, including base measurements (height and weight) and circumferences (waist and hip). All measurement data was obtained using standardised techniques and calibrated equipment [10]. The WC measurement was taken to the nearest 0.1 cm using a Lufkin executive thin line tape measure (Lufkin W606PM) (Apex Tool Group, USA) at the narrowest point between the lower costal border and the superior iliac crest perpendicular to the long axis of the trunk. The hip circumference (HC) was taken at the greatest posterior protuberance of the gluteal muscles also to the nearest 0.1 cm.

Blood pressure measurements were taken using an aneroid sphygmomanometer (Erka, Perfect Aneroid Clinic 48, Germany) after a rest period of five minutes in a sitting position. Systolic and diastolic blood pressure was measured twice, at least 30 min apart, when participants presented with a very high or low blood pressure, and the mean of these two measurements recorded. Blood samples, for serum insulin, glucose and a fasting lipogram, were drawn following an overnight fast of ten hours.

### Criteria for metabolic syndrome classification

For a diagnosis of the MetS, participants had to display two of the four abnormal criteria with the inclusion of a raised WC according to the International Diabetes Foundation (IDF) [11]. The following criteria included: (a) a compulsory raised WC of ≥94 cm for men and ≥80 cm for women; (b) elevated triglycerides (TG): >1.7 mmol/l; (c) low high-density lipoprotein-cholesterol (HDL-c) of <1.03 mmol/l for men and <1.3 mmol/l for women; (d) raised blood pressure (BP) ≥130/85 mmHg or on hypertension treatment; and (e) raised fasting blood glucose (FBG) concentration > 5.6 mmol/l or on diabetes treatment.

### Statistical analysis

Statistical analyses were performed using Statistica version 12 software (Statsoft, Tulsa OK, USA). The means and standard error of the mean (SEM) were calculated for all parameters, and factorial analysis of variance (ANOVA) employed to evaluate differences between various groups. The Bonferroni *post hoc* test was subsequently used to assess the significance of differences found between groups. Spearman correlations were used to determine associations between variables. *P*-values of ≤ 0.05 were considered statistically significant and the mean ± SEM was used for all results reported.

## Results

### Metabolic syndrome-specific participant characteristics and classifications

From the total *n* = 191 participants, three participants withdrew their consent because of time constraints (*n* = 2 women and *n* = 1 male). One hundred and forty seven women (78%) and 41 men (22%) were recruited for this study which took place between April – June 2015. The mean age of all participants was 38.78 ± 0.78 years (37.81 ± 0.87 in women and 42.27 ± 1.68 years in men).

**Fig. 1:**
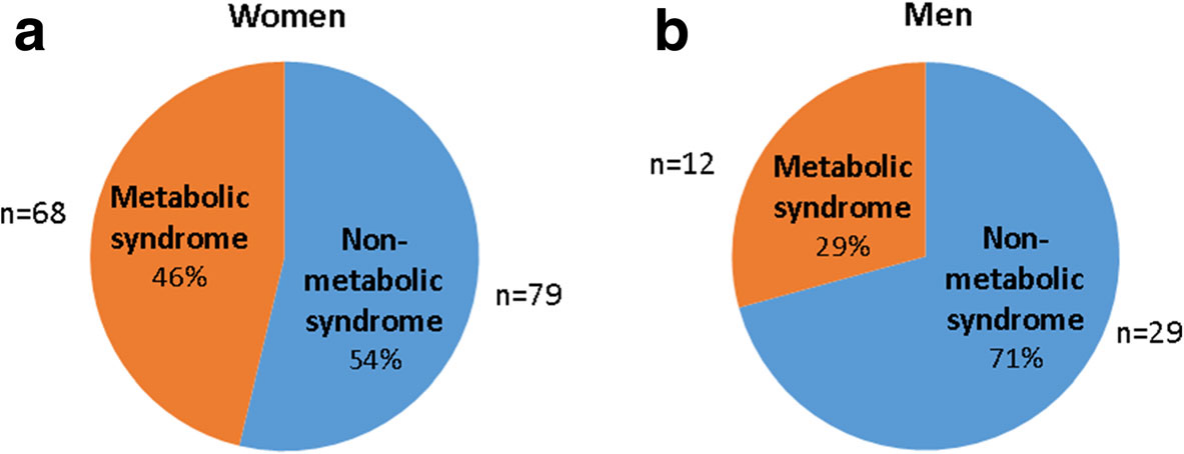
Prevalence of the metabolic syndrome in men and women according to IDF criteria

**Fig. 2:**
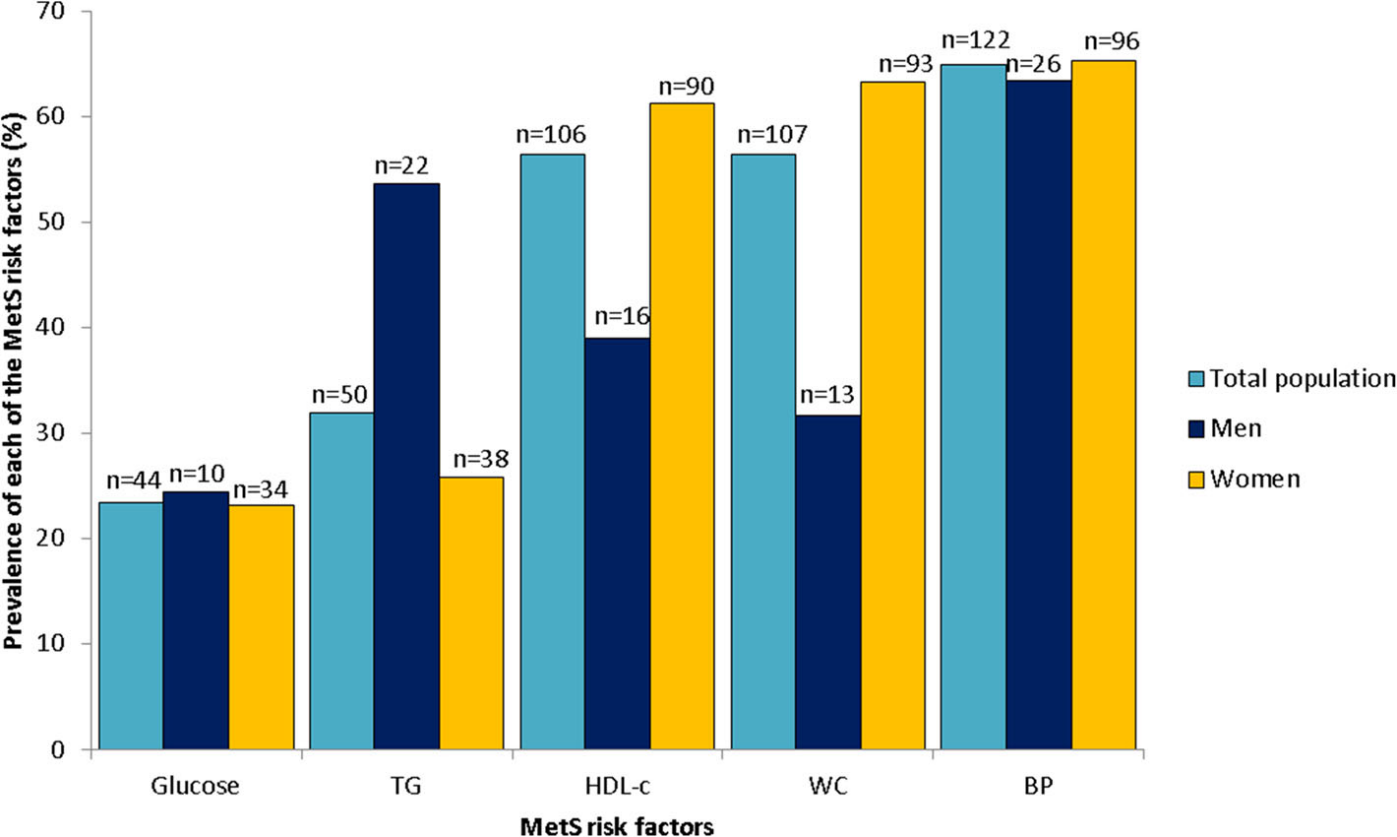
Prevalence of each component of the metabolic syndrome in the total population, men, as well as women

**Fig. 3:**
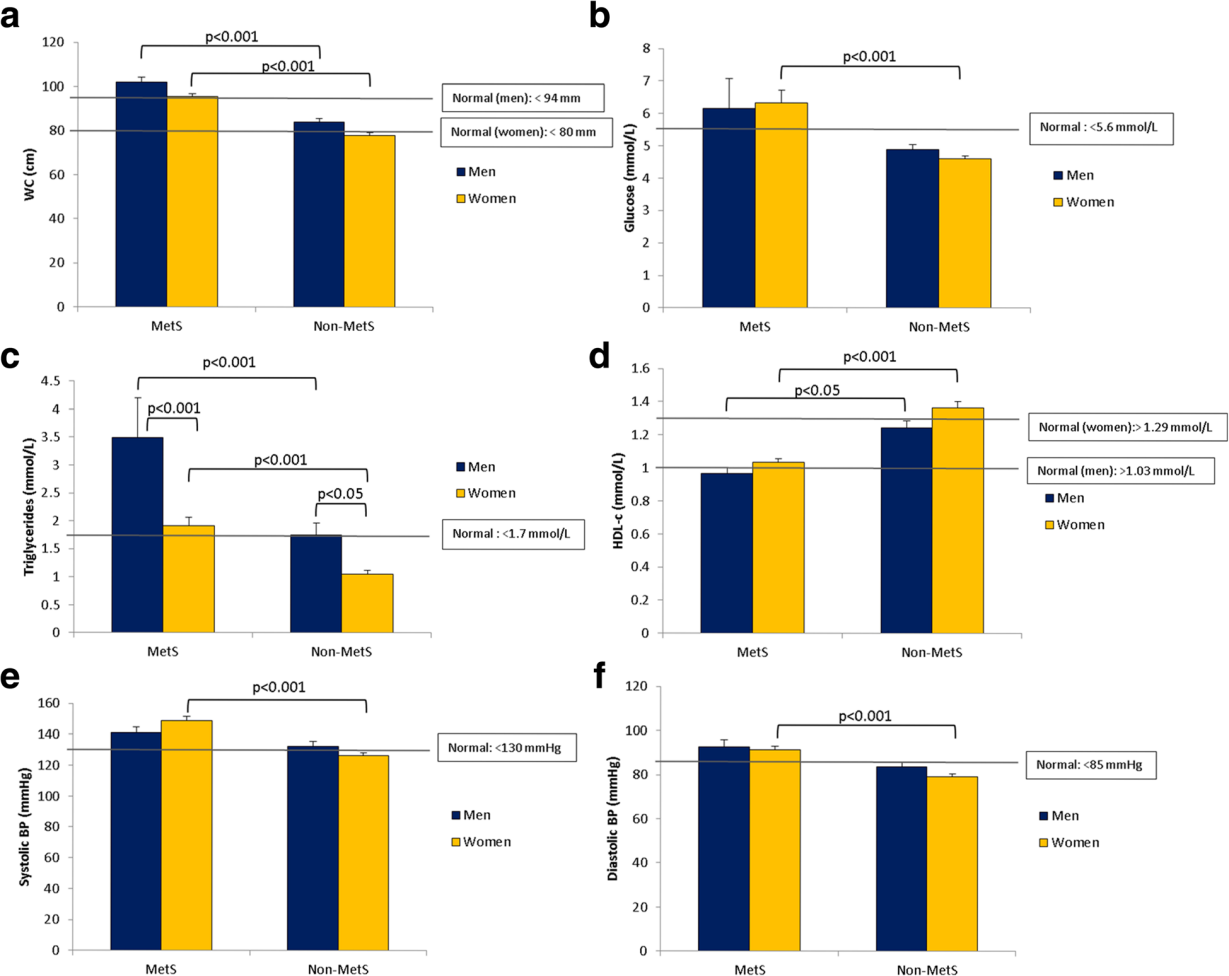
Risk factors according to the IDF criteria for men and women in the MetS and non-MetS groups for (**a**) waist-circumference; (**b**) glucose; (**c**) triglycerides, (**d**) HDL-c; (**e**) systolic BP and (**f**) diastolic BP. The grey solid lines represent the normal cut-off values according to the IDF

Overall, the prevalence of the MetS (based on the IDF criteria) was 42.6% (46.3% in women and 29.3% in men) (Fig. 1). The prevalence of each component of the MetS in this population was: 56.4% for abdominal obesity as measured by WC (63.3% in women; 31.7% in men), 23.4% for elevated FBG (23.1% in women; 24.4% in men), 31.9% for elevated TG level (25.9% in women; 53.7% in men), 56.4% for low HDL-c level (61.2% in women; 39.0% in men), and 64.9% for hypertension (65.3% in women; 63.4% in men) (Fig. 2). Statistical significant differences were observed for all the indices of the MetS as displayed in Fig. 3. For WC, both male and female participants in the MetS group had a significantly higher WC compared to their non-MetS counterparts (*p* < 0.001 for both). Higher FBG levels were also observed in the MetS groups, but only significantly higher for the MetS women (*p* < 0.001). Similar observations were also made for systolic and diastolic BP. Significant differences were observed between all groups for TG levels. Not only did the male and female MetS participants display significantly elevated TG levels compared to their non-MetS counterparts (*p* < 0.001), but interestingly enough, men showed significantly higher TG levels compared to the women in both the MetS (*p* < 0.001) and non-MetS (*p* < 0.05) groups. High-density lipoprotein-cholesterol were also significantly higher in the non-MetS groups compared to their MetS counterparts in both genders (*p* < 0.001 for women and *p* < 0.05 for men).

Although the IDF uses WC as the first risk factor for categorising a person with the MetS, we were somewhat concerned regarding the health implications of obesity at the hand of body mass index (BMI). When men and women were classified into the different BMI classes (Table 1), we found that about 40% of the MetS men were overweight, 40% were obese class I and about 20% obese class III, whereas the MetS women were almost equally distributed between the overweight, obese class I, obese class II and obese class III categories. Of particular concern were the participants in the non-MetS groups, especially the women with approximately 61% of them having a BMI of higher than 25 kg/m^2^ compared to 41% of the men without the MetS.

**Table 1:** Distribution of participants into different BMI classes according to the MetS status and gender

BMI range	BMI classification	MetS+ Men	MetS+ Women	MetS- Men	MetS- Women	TOTAL
<18.5	Underweight	0	0	0	2	2
18.5–24.9	Normal	0	0	17	28	45
25–29.9	Overweight	5	17	11	36	69
30–34.9	Obese class I	5	20	1	3	29
35–39.9	Obese class II	0	17	0	7	24
>40	Obese class III	2	14	0	3	19
TOTAL		*n* = 12	*n* = 68	*n* = 29	*n* = 79	*n* = 188

**Fig. 4:**
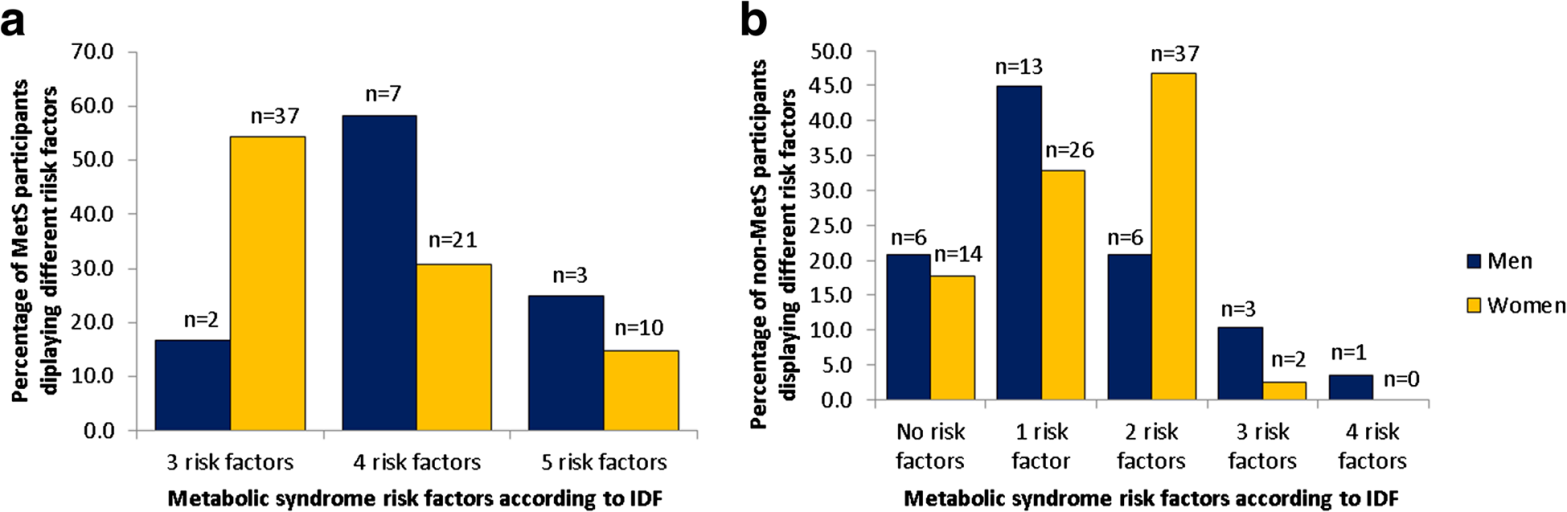
Distribution of risk factors according to the IDF criteria for participants diagnosed with (**a**) the metabolic syndrome (**b**) and those without the metabolic syndrome

**Fig. 5:**
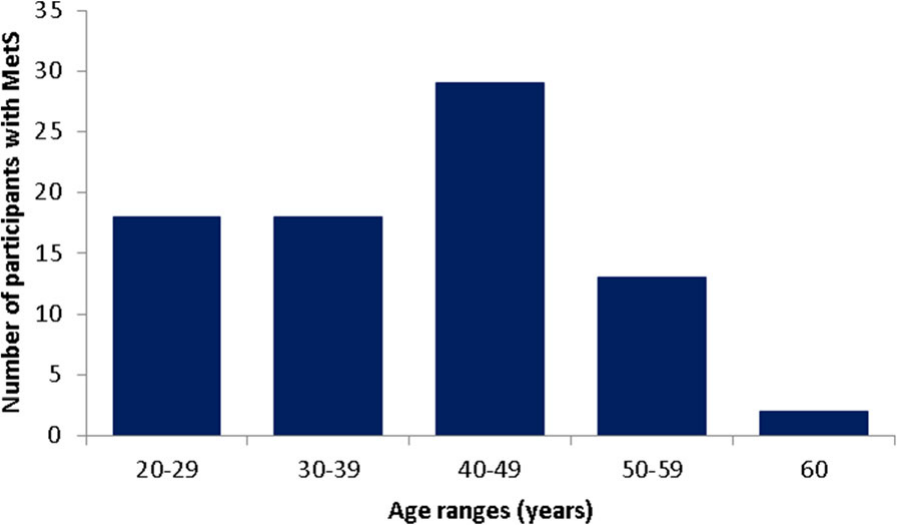
Distribution of participants with the MetS according to age

Almost half (48.8%) the MetS population presented with three risk factors, 35.0% with four and 16.2% with all five risk factors (Fig. 4a). Figure 4b displays individuals who have zero and up to four risk factors with the exception of WC as per IDF criteria. The data also indicates more participants were categorized as having the MetS between the age range 40–49 years (Fig. 5).

### Correlations for relationships between body mass indices and MetS risk factors

**Fig. 6:**
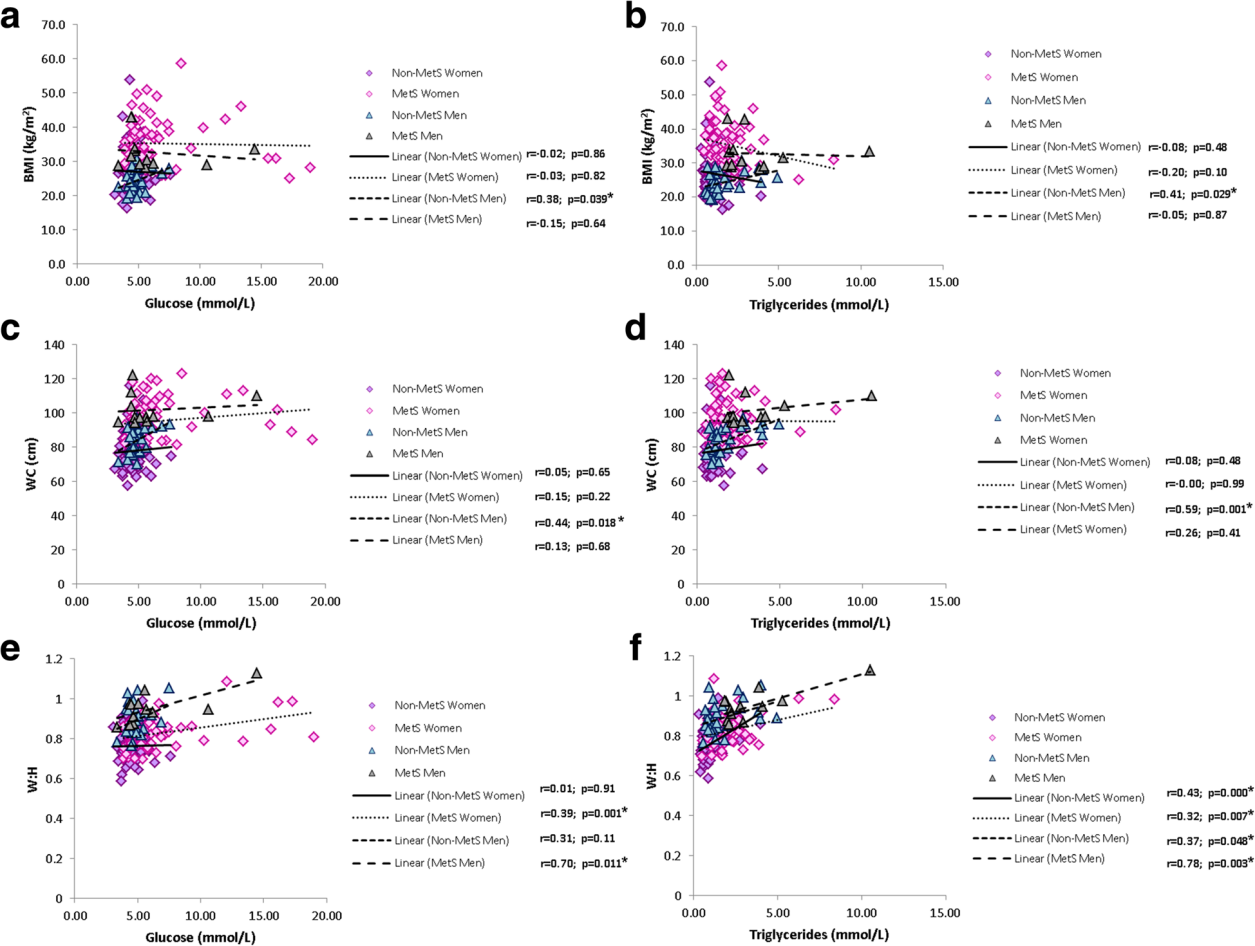
Correlations showing relationships between body fat indices and the MetS risk factors: **a** BMI and glucose, (**b**) BMI and triglycerides, (**c**) WC and glucose, (**d**) WC and triglycerides, (**e**) W:H and glucose and (**f**) W:H and triglycerides. Significance are indicated (*) next to the respective groups

To further describe the interactions between the risk factors of the MetS, correlations were performed which indicated significant associations between the three obesity parameters; BMI, WC and W:H, and the MetS risk factors, FBG and TG. The majority of significant associations were evident either for the non-MetS men non-MetS group or the MetS women. The non-MetS male group showed significant associations when both BMI and WC were correlated with FBG (*p* = 0.039 for BMI and *p* = 0.018 for WC) and TG (*p* = 0.029 for BMI and *p* = 0.001 for WC) (Fig. 6). Although only the non-MetS men displayed significant, but moderate, correlations as seen in Fig. 6a-d, both MetS women and men showed significant correlations between W:H and FBG (Fig. 6e), whereas all groups showed a positive association between W:H and TG (Fig. 5f).

**Fig. 7:**
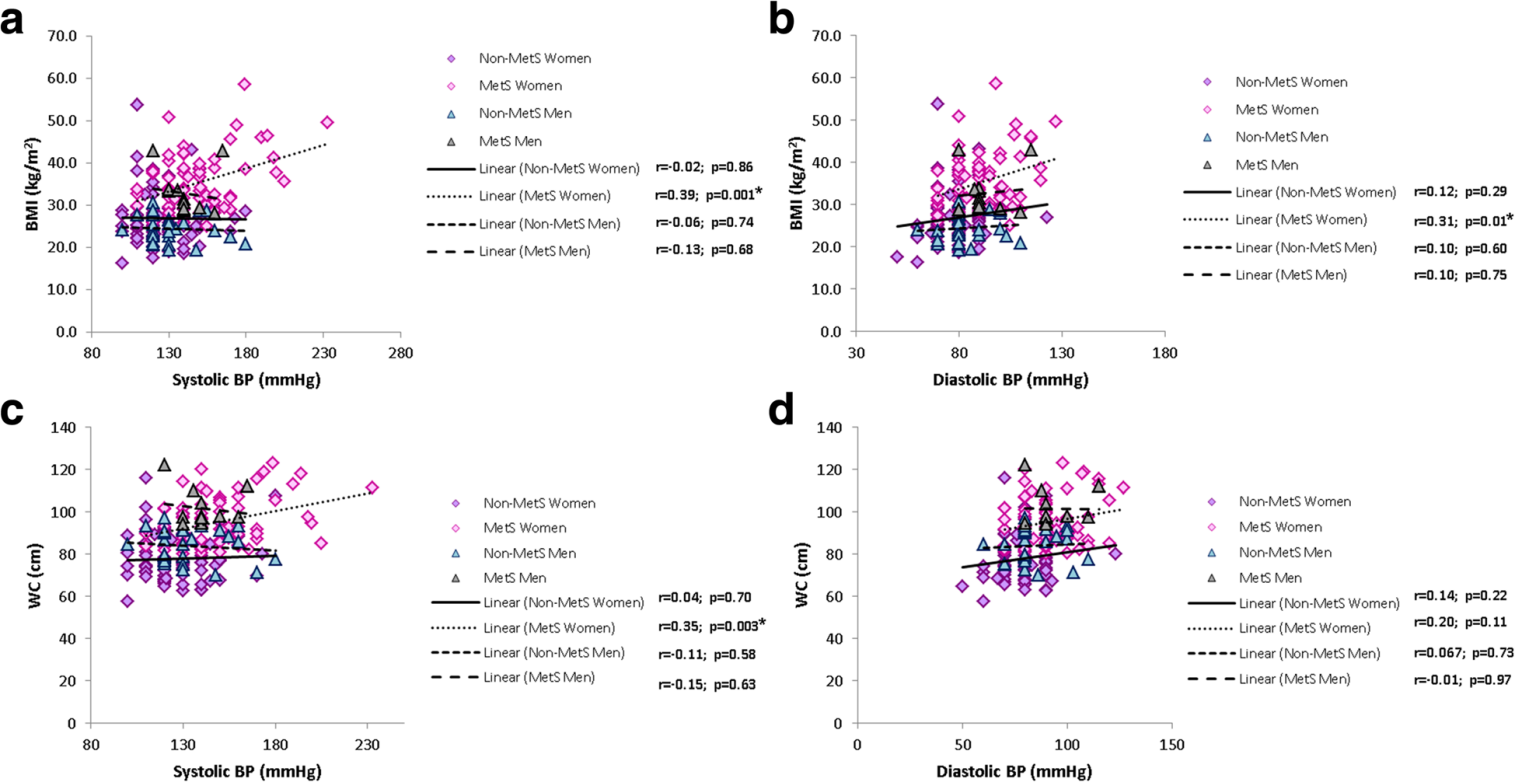
Correlations showing relationships between BMI and systolic and diastolic BP (**a** and **b** respectively) and WC and systolic and diastolic BP (**c** and **d**). Significance are indicated (*) next to the respective groups

Women in the MetS group’ BMI were significantly, but moderately, correlated with systolic (*p* = 0.001) and diastolic BP (*p* = 0.01). However, W:H was not significantly correlated to either systolic or diastolic BP, whereas WC was only correlated with systolic BP (*p* = 0.003) in the female MetS group (Fig. 7).

We also calculated a MetS risk score (RS) for all groups either without WC (value out of four) or with WC (value out of five), with the presence of a risk factor accounting for one RS point. These risk scores were then correlated with the three obesity indices (Table 2). In the MetS men a strong negative correlation (*p* = 0.01) between the MetS RS and BMI was shown, whereas the non-MetS men (moderate positive correlation) and the MetS women (weak positive correlation) showed significant associations with WC. A moderate positive correlation was also evident in the MetS female group for W:H (*p* = 0.008). For non-MetS women, a weak positive correlation was only evident when WC was taken into consideration in the correlation between the MetS RS and both WC (*p* = 0.011) and W:H ratio (*p* = 0.032) with a trend for BMI (*p* = 0.054).

**Table 2:** Correlations between the MetS risk scores (RS) and the obesity indices BMI, WC and W:H

	Parameters	BMI (kg/m2)	WC (cm)	W:H
MetS Men	MetS RS without WC (*n* = 12)	–0.71 (*p* = 0.01)	–0.47 (*p* = 0.12)	0.39 (*p* = 0.21)
	MetS RS with WC (*n* = 12)	–0.71 (*p* = 0.01)	–0.47 (*p* = 0.12)	0.39 (*p* = 0.21)
Non-MetS Men	MetS RS without WC (*n* = 29)	0.29 (*p* = 0.12)	0.45 (*p* = 0.013)	0.35 (*p* = 0.066)
	MetS RS with WC (*n* = 29)	0.36 (*p* = 0.054)	0.51 (*p* = 0.005)	0.37 (*p* = 0.052)
MetS Women	MetS RS without WC (*n* = 68)	0.12 (*p* = 0.32)	0.27 (*p* = 0.029)	0.32 (*p* = 0.008)
	MetS RS with WC (*n* = 68)	0.12 (*p* = 0.32)	0.27 (*p* = 0.029)	0.32 (*p* = 0.008)
Non-MetS Women	MetS RS without WC (*n* = 79)	–0.20 (*p* = 0.073)	–0.20 (*p* = 0.08)	0.023 (*p* = 0.84)
	MetS RS with WC (*n* = 79)	0.22 (*p* = 0.054)	0.28 (*p* = 0.011)	0.24 (*p* = 0.032)

## Discussion

The prevalence of the MetS is globally on the rise, and studies have shown that this is also the case for Africa. A recent study by Hoebel et al. [12] showed that Africans have the highest prevalence of the MetS compared to Caucasians. Other African-based studies were also able to confirm findings of a higher prevalence of the MetS in South Africa [7, 13], compared to those from other areas of the world [14–16] and even smaller African countries themselves such as Nigeria, Ethiopia, Cameroon, Angola and Tunisia [17–22].

The influence of gender on the development and characterisation of the MetS have not been that well documented, since the overall prevalence of the MetS, irrespective of gender, have always been the main concern. However, the ever increasing prevalence of the MetS, especially amongst the female population, is now starting to gain much needed attention [7, 8]. This also holds true for our specific population, since more women were characterised as having the MetS than men.

According to the JIS definition, the prevalence of the MetS in an urban black population of Cape Town was 44.9% in women and 17.3% in men, after adjusting for age [13]. These statistics were somewhat similar to that found in our population for both women (46.3%) and men (29.3%), with the exception of using the IDF definition. The prevalence of the MetS found by Peer et al. [13] was much higher than that reported by Motala et al. [6], which also used the JIS definition in an urban population. They showed that the prevalence of the MetS increased with age [6], however for our study participants, a highest prevalence of the MetS was noted between the ages of 40–49 years, followed by a decrease thereafter.

Similar to our study population, a US-based national health survey study (NHANES III, 1988–1994) and the study by Peer et al. [13] indicated that abdominal obesity, elevated TG and low HDL-c was the most prominent risk factors in women [13, 23]. Both these studies, as well as our study used different diagnostic criteria for the MetS with similar outcomes. In our female population, BP was the most dominant feature (65.3%), followed closely by WC (63.3%) and HDL-c (61.2%). These values are lower than those reported by Peer et al. [13] (central obesity, 86% and low HDL-c, 75%), but it is expected since they used the JIS and not the IDF definition. Although the high occurrence of these risk factors could possible explain the increased prevalence of the MetS in our female population, this might not necessarily be the case for the male population since we cannot rule out the fact that the relatively low number of men who partook in the study could have contributed to the relatively low prevalence of MetS in the men. Irrespective, the following three individual risk factors also occurred quite frequently in the men: elevated BP (63.4%), increased TG (53.7%) and lower HDL-c (39.0%). Similar to our findings, the NHANES study also concurred that the combination of increased TG, low HDL-c (22), and hypertension was most frequent (18.0%), whereas hypertension was the only prevalent parameter in the study by Peer et al. [13].

Statistical significant gender differences were observed for all indices of the MetS in our study. Almost all the MetS risk factors were significantly different between men and women of the MetS and non-MetS groups with the exception of FBG and systolic and diastolic BP. Only TGs showed differences between men and women of the respective groups, which could be attributed to their testosterone (T) levels. This is quite likely since a study by Grosman et al. [24] found a significant but weak negative correlation between TG levels and T concentrations. In the aforementioned study, the prevalence of the MetS also increased as T levels became lower, suggesting that the men with the MetS will have lower T levels and in turn higher TG levels. Furthermore, the higher incidence of the MetS in subjects with lower T levels was also associated with abdominal obesity [25–29]. Kaplan et al. [30] for example examined T levels in *n* = 864 subjects with and without the MetS and found that obese men with the MetS had significantly lower T levels than non-obese men with or without the MetS. Considering our findings that the men with the MetS had higher TG levels compared to the women with the MetS, as well as the men in the non-MetS group (i.e. those without abdominal obesity), these results corroborates that found in the studies mentioned above. Although total T might partially explain why TG’s are elevated in men with the MetS, estrogen (E2) should also be considered since around 60% of circulating E2 in men is produced by aromatisation of T [31, 32]. A recent experimental study showed that, independent of T, body fat increased when E2 levels were lower in men. Also, even though both T and E2 are associated with variations in body composition in women and men [33, 34], a study by Antonio et al. [35] found E2 not to be associated with the development of the MetS in their male population. Here, instead, lower E2/T ratio, which reflects less aromatisation of T to E2, was associated with a 62% reduced risk of incident MetS, and was dependent on body fat. This study further showed that lower T was associated with higher TG, whereas a lower E2/T ratio was associated with lower TG. Although these factors were associated with body fat, total E2 was only associated with higher TG levels and not body fat. This indicates that changes in body composition may modify the association between the E2-androgen balance and MetS, which may account for discrepancies in the literature regarding E2 and obesity. This fact is in contrast to a study by Wang et al. [36] which showed that obesity was associated with increased E2 in men, as well as animal experiments in which a lack of E2 resulted in increased body fat [37]. Considering our study population and the fact that participants were either overweight, obese or had normal body weight, complicates the current understanding of E2’ effect on TGs and body weight. It is therefore highly recommended not only measuring E2 and T, but rather the E2/T ratio in order to fully elucidate the relationship between sex hormones, obesity and the MetS.

Although literature states that the prevalence of the MetS differs by age, ethnicity and gender, the population being studied, as well as the definition used, can also significantly adjust the prevalence. What also further complicates this issue is the fact that the syndrome is only diagnosed whenever three out of the five risk factors are present, which differs according to the definition used. This is especially accurate for the African population since the different criteria for diagnosing the MetS have not been adapted for either the South African or African populations [38]. One of the key contributors to the MetS is WC, and as of yet, no clearly defined ethnic-specific WC cut-off points have been developed for Africans, and this may directly impact on the classification of the MetS, as it can either over- or underestimate the prevalence of the MetS in this context. It should also be noted that an African-based MetS definition with specific cut-off values for the different risk factors should be developed in order to accurately estimate the occurrence of the MetS.

Recent studies have suggested WC and BP cut-off points for various African populations [6, 39–41], however, more epidemiological studies are needed to confirm that these suggested cut-off points can actually be used with confidence in the African population. Peer et al. [13] reported optimal WC cut-off values based on a black population from Cape Town (83.9 cm men and 94 cm women) and confirmed the findings from Motala et al. (2011) [6]. However, these cut-off values differ from what is currently recommended for Africans [11, 42]. The studies by Peer et al. [13] and Motala et al. [6] furthermore suggested that current WC cut-off values for Africans may require change or adaptations depending on the population used and the specific setting.

We are also aware that genetic background, diet, levels of physical activity, and levels of over- or undernutrition also influence the prevalence of both the syndrome and its components. These issues need to be addressed in order to fully understand the complexity of the syndrome, as well as to enable proper intervention and treatment programmes.

Our study also needs to recognise some limitations such as the nature of the study design. The results reported here cannot be generalized to the entire population, and is limited in its ability to elucidate a causal relationship between associations. The response rate for the men participating was very low (22.3%). This introduces the possibility of a skewed data representation and further limits the generalizability of the findings. No South-African/African-based definition of the MetS with specific cut-off values exist, which makes an accurate estimation of the prevalence of the MetS in this population difficult. Although dietary and physical activity data was gathered the data could not be analysed due to poor reporting from participants.

## Conclusion

Evidence shows that the prevalence of the MetS is not only of global concern, but it is also alarmingly high in the South African population. This study revealed that the prevalence of the MetS is indeed high in a farm workers community of both genders in the Western Cape of South Africa. This therefore highlights the importance of developing ethnic-based definitions with clear and different cut-off values for Africans specifically. Our study also confirmed that the women might be more at risk of developing the MetS compared to the men. Future studies should therefore not only focus on developing specific criteria for Africans, but should also focus every effort to make individuals aware of the risk factors and the use of proper therapeutics or other nutritional or exercise interventions for the treatment of the MetS. It is also important to consider the cardio-metabolic burden as a consequence of the ever increasing prevalence of the MetS and its risk factors.

## Abbreviations

ANOVA: Analysis of variance
BMI: Body mass index
BP: Blood pressure
FBG: Fasting blood glucose
HDL-c: High-density lipoprotein-cholesterol
IDF: International diabetes federation
JIS: Joint interim statement
MetS: Metabolic syndrome
RS: Risk score
SEM: Standard error of the mean
TG: Triglycerides
WC: Waist circumference
W:H: Waist to hip ratio
